# Neoadjuvant chemoradiotherapy versus immunochemotherapy in esophageal squamous cell carcinoma: a propensity-matched analysis of response, survival, lymph node pattern, and immune microenvironment

**DOI:** 10.3389/fimmu.2026.1862815

**Published:** 2026-07-29

**Authors:** Zhou Yehan, Xiaoqing Zhou, Shangzhi Hu, Qiao Yang, Xuefeng Leng, Wenwu He, Qifeng Wang, Jiahua Lyu, Haomiao Qing, Yang Liu, Hong Yang

**Affiliations:** 1 Department of Pathology, Sichuan Clinical Research Center for Cancer, Sichuan Cancer Hospital & Institute, Sichuan Cancer Center, University of Electronic Science and Technology of China, Chengdu, China; 2 Department of Endoscopy, Sichuan Clinical Research Center for Cancer, Sichuan Cancer Hospital & Institute, Sichuan Cancer Center, University of Electronic Science and Technology of China, Chengdu, China; 3 Department of Thoracic Surgery, Sichuan Clinical Research Center for Cancer, Sichuan Cancer Hospital & Institute, Sichuan Cancer Center, University of Electronic Science and Technology of China, Chengdu, China; 4 Department of Thoracic Radiation Oncology, Sichuan Clinical Research Center for Cancer, Sichuan Cancer Hospital & Institute, Sichuan Cancer Center, University of Electronic Science and Technology of China, Chengdu, China; 5 Department of Radiology, Sichuan Clinical Research Center for Cancer, Sichuan Cancer Hospital & Institute, Sichuan Cancer Center, University of Electronic Science and Technology of China, Chengdu, China

**Keywords:** esophageal squamous cell carcinoma, immune microenvironment, neoadjuvant chemoradiotherapy, neoadjuvant immunochemotherapy, prognosis

## Abstract

**Objective:**

To compare efficacy, survival and tumor immune microenvironment between neoadjuvant chemoradiotherapy (NCRT) and neoadjuvant immunochemotherapy (NICT) in patients with esophageal squamous cell carcinoma (ESCC) to guide individualized treatment.

**Methods:**

A total of 346 locally advanced ESCC patients after 1:1 propensity score matching were retrospectively analyzed. Pathological, radiological and multiplex immunofluorescence assessments were performed, and survival outcomes were evaluated.

**Results:**

NCRT resulted in significantly greater tumor volume reduction (22.09 vs. 15.37 mm³), lower residual primary tumor ratio (14.10% vs. 32.23%), higher tumor downstaging and pathological complete response (pCR) rates (all p < 0.05). NCRT showed more prominent lymph node regression adjacent to the primary tumor than in peripheral nodes. Overall survival (OS) and recurrence-free survival (RFS) were comparable between groups. Among pCR patients, NICT achieved significantly better OS (p = 0.0051) and RFS (p = 0.018), while NCRT was associated with superior RFS in non-pCR patients (p = 0.0099). In the NICT group, high PD-L1 expression and low tumor burden tended to correlate with pCR. Immune microenvironment analysis revealed fewer immunosuppressive T cells and immune-evasive tumor cells in the NICT group.

**Conclusion:**

NCRT presents superior local tumor regression, while NICT benefits pCR patients by optimizing the immune microenvironment. High PD-L1 expression and low tumor burden favor NICT. These findings support individualized neoadjuvant strategy selection for ESCC.

## Introduction

Esophageal squamous cell carcinoma (ESCC) accounts for the majority of esophageal cancer cases in East Asia, and locally advanced disease remains a major therapeutic challenge. Based on the landmark CROSS and 5010 studies (1, 2), neoadjuvant therapy has been consistently demonstrated to achieve remarkable tumor downstaging, improve radical resection rates, and prolong survival, thereby establishing itself as the standard of care for locally advanced esophageal squamous cell carcinoma.

Current neoadjuvant strategies include chemotherapy (single-agent or combination), neoadjuvant chemoradiotherapy (NCRT), and neoadjuvant immunochemotherapy (NICT). Among these, NCRT has long been the traditional standard recommended by international clinical guidelines. With the advent of immune checkpoint inhibitors, emerging evidence from trials such as ESCORT-NEO (3) has validated the efficacy and safety of NICT, supporting its promising role in the neoadjuvant setting. Prospective phase 2 studies, including NIC-ESCC2019 and KEEP-G 03, have further demonstrated encouraging pathological responses and manageable safety profiles with neoadjuvant immunochemotherapy (4, 5). Consequently, NICT has emerged as a strong competitor to conventional NCRT, and the comparative advantages between these two approaches have become a highly debated clinical hotspot (6, 7).

Previous studies suggested that NICT may be preferred by surgeons owing to fewer adverse events, absence of radiation-induced tissue adhesions and fibrosis (8). In contrast, radiation oncologists often emphasize the lower pathological complete response (pCR) rate associated with NICT relative to NCRT (9). Furthermore, whether pCR can reliably translate into superior long-term prognosis remains controversial across different neoadjuvant platforms (10).

To address these critical uncertainties, the present study retrospectively analyzed two well-balanced cohorts of patients with locally advanced esophageal squamous cell carcinoma treated with NCRT or NICT. By comprehensively comparing post-treatment pathological changes, short-term treatment responses, long-term survival outcomes, and tumor microenvironment characteristics, we aimed to identify the distinct advantages and differential effects of the two regimens. We hope our findings will provide evidence-based insights for individualized neoadjuvant strategy selection in clinical practice.

## Study subjects and methods

### Study subjects

This study retrospectively reviewed 3,218 patients with esophageal cancer treated at our institution between December 2018 and December 2022. The following inclusion and exclusion criteria were applied: (1) diagnosis of primary locally advanced esophageal squamous cell carcinoma; (2) absence of concurrent malignant tumors; (3) receipt of preoperative neoadjuvant therapy, including radiotherapy plus chemotherapy or chemotherapy combined with immunotherapy; (4) subsequent radical esophagectomy after neoadjuvant therapy; (5) no clinical evidence of distant metastasis; and (6) no history of autoimmune diseases.

**Figure 1:**
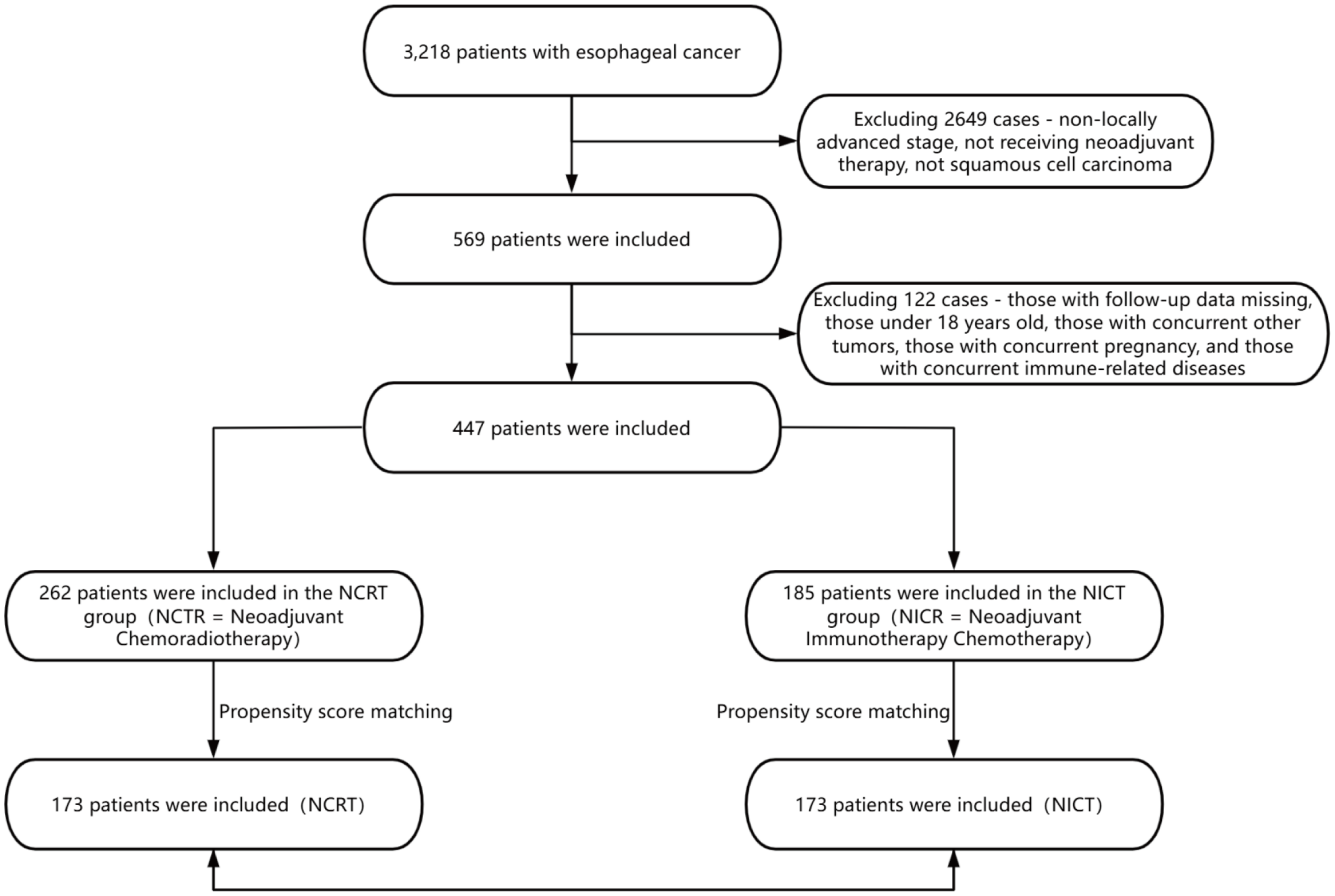
Patient selection and study flow diagram. A flowchart depicting the enrollment, propensity score matching (PSM), and final cohort allocation of esophageal cancer patients receiving neoadjuvant chemoradiotherapy (NCRT) or neoadjuvant immunochemotherapy (NICT).

Initially, 2,649 cases were excluded due to non-locally advanced disease, absence of neoadjuvant therapy, or non-squamous histology, leaving 569 eligible patients. After further exclusion of 122 patients owing to missing follow-up data, age <18 years, coexisting tumors, pregnancy, or immune-related disorders, 447 patients were included for analysis. Among them, 262 patients received neoadjuvant chemoradiotherapy (NCRT) and 185 received neoadjuvant immunochemotherapy (NICT).To minimize selection bias and balance baseline characteristics between the two groups, propensity score matching (PSM) was performed. After matching, 173 patients remained in each group (NCRT and NICT) for subsequent comparative analyses of treatment outcomes and resistance mechanisms (**Figure 1**).

Neoadjuvant Treatment Regimens: NCRT Group: Patients received concurrent chemoradiotherapy combined with immunotherapy. The chemotherapy regimens involved either monotherapy or combination therapy, including agents such as albumin-bound paclitaxel, carboplatin, cisplatin, docetaxel, oxaliplatin, and fluorouracil, with a median of 1.99 ± 0.52 cycles administered. Concurrent image-guided intensity-modulated radiotherapy (IMRT) was delivered with the following fractionation: 2.0 Gy per fraction to the primary gross tumor volume (GTV), 2.0 Gy per fraction to the left and right lymph node target volumes (GTV-InL and GTV-InR, respectively), and 1.8 Gy per fraction to the clinical target volume (CTV). Treatment consisted of 20.32 ± 1.14 fractions, yielding a total radiation dose of 40.49 ± 1.75 Gy. Immunotherapy agents included nivolumab, pembrolizumab, or sintilimab; NICT Group: Patients received immunotherapy combined with chemotherapy, without radiotherapy. The chemotherapy regimens were identical to those used in the NCRT group, encompassing the same single or combination agents, and were administered for a comparable number of cycles. Immunotherapy likewise consisted of nivolumab, pembrolizumab, or sintilimab.

### Methods

Tissue samples were processed following standard histological protocols. Briefly, surgically resected tumor specimens were fixed in 10% neutral buffered formalin, paraffin-embedded, and sectioned for routine hematoxylin and eosin (H&E) staining. The stained sections were then subjected to pathological evaluation.

Primary tumor tumor regression grade (PT-TRG) was assessed according to the American Joint Committee on Cancer/College of American Pathologists (AJCC/CAP) guidelines, while lymph node tumor regression grade (LN-TRG) was evaluated based on our previously established criteria (**Supplementary Figure 1**) (11). Both grading systems primarily consider the proportion of residual viable tumor cells relative to the tumor bed or stromal tissue. All histological assessments were performed independently by two experienced pathologists. In cases of discrepancy, immunohistochemical staining was applied for further clarification, and a senior pathologist was consulted to review and finalize the results, thereby ensuring accuracy and consistency. For radiographic assessment, tumor volume was calculated using the following formula: Tumor volume of esophageal cancer = Sum of the tumor cross-sectional areas × Slice thickness.

Cumulative distribution step plots were used to compare post-treatment tumor volume change, residual primary tumor, and residual lymph node tumor rate between the NCRT and NICT groups. Sankey diagrams illustrated the shifts in T and N staging before and after treatment. Anatomical lymph node heatmaps were employed to compare post-treatment lymph node tumor residual rate, lymph node positivity probability, and the distribution of positive lymph nodes between the two groups.

Multiplex immunofluorescence (mIF) was performed on post-treatment specimens from 38 patients (NICT: 30, NCRT: 8). This imbalance arose from quality-control exclusions and funding constraints, making the analysis exploratory and underpowered; results should be interpreted with caution. To investigate the regulatory differences between neoadjuvant immune chemotherapy (NICT) and neoadjuvant chemoradiotherapy (NCRT) on immune cell subsets and PD-L1 expression in the esophageal cancer tumor microenvironment, 38 patients (30 NICT, 8 NCRT) were enrolled for multiplex immunofluorescence (mIF)-based microenvironment analysis. mIF staining was performed using the Opal system (Akoya Biosciences) with antibodies against CD3 (Opal 520), CD68 (Opal 570), CD20 (Opal 620), PD-L1 (Opal 690), and CK-pan (Opal 780), followed by DAPI (Opal 480) counterstaining; antibody stripping via microwave heating was applied after each cycle to eliminate cross-reactivity. High-resolution mIF images were acquired using a Leica Aperio VERSA whole-slide scanner, with regions of interest (ROIs, encompassing tumor and tumor bed regions) manually annotated on each section. Images were imported into the Halo digital pathology platform (Indica Labs), where the “Multiplex Immunofluorescence” module enabled cell segmentation and phenotyping based on DAPI staining and fluorescence intensity thresholds; core phenotypes (CD3+, CD20+, CD68+, CD3+PD-L1+, CK-pan+PD-L1+, CD68+PD-L1+) were quantified for density (cells/mm²), positive rate (%), and absolute count.

### Statistical analysis

This study employed Cox regression, Kaplan-Meier curves, and log-rank tests to analyze recurrence-free survival (RFS) and overall survival (OS). Statistical analyses were conducted using the R software (http://www.R-project.org, The R Foundation) and the Free Statistics analysis platform. RFS was defined as the time from surgery to recurrence or death from any cause, while OS was defined as the time from surgery to death. A p value of <0.05 was considered statistically significant.

## Results

Prior to matching, significant imbalances were observed in several baseline characteristics between the two groups, including tumor location and clinical T and N stages, as indicated by standardized mean differences (SMD) > 0.1. These differences were effectively balanced through 1:1 propensity score matching (PSM), thereby minimizing selection bias and ensuring comparability in demographic and tumor staging variables for subsequent analyses (**Supplementary Figures 1A–C**, **Table 1**). Among the 346 patients with esophageal cancer included in this study, 300 (86.71%) were male and 46 (13.29%) were female. The mean age was 61.09 years (median 62 years, range 42–76 years). The majority of tumors were located in the lower esophagus (53.47%, n = 185). The median follow-up time was 24.23 months.

**Table 1:** Clinical baseline conditions before and after propensity score adjustment.

	Unmatched			Propensity score matched		
Item	NCRT	NICT	SMD	NCRT	NICT	SMD
n	262	185		173	173	
Sex(M)	220 (84.0)	161 (87.0)	0.087	150 (86.7)	150 (86.7)	0.001
Age	60.29 (7.17)	61.19 (7.33)	0.124	61.02 (7.03)	61.16 (7.33)	0.02
Smoking	177 (67.6)	136 (73.5)	0.131	134 (77.5)	128 (74.0)	0.081
Drinking	172 (65.6)	124 (67.0)	0.029	126 (72.8)	118 (68.2)	0.102
Location			0.193			0.037
Upper	39 (14.9)	17 (9.2)		16 (9.2)	16 (9.2)	
Middle	101 (38.5)	69 (37.3)		66 (38.2)	63 (36.4)	
Lower	122 (46.6)	99 (53.5)		91 (52.6)	94 (54.3)	
cT			0.344			0.028
1	0 (0.0)	2 (1.1)		0 (0.0)	0 (0.0)	
2	19 (7.3)	4 (2.2)		4 (2.3)	4 (2.3)	
3	220 (84.0)	171 (92.4)		161 (93.1)	162 (93.6)	
4	23 (8.8)	8 (4.3)		8 (4.6)	7 (4.0)	
cN			0.303			0.045
0	6 (2.3)	17 (9.2)		6 (3.5)	7 (4.0)	
1	95 (36.3)	59 (31.9)		60 (34.7)	57 (32.9)	
2	131 (50.0)	88 (47.6)		86 (49.7)	88 (50.9)	
3	30 (11.5)	21 (11.4)		21 (12.1)	21 (12.1)	

A comparative analysis between the NCRT and NICT groups revealed no statistically significant differences (p ≥ 0.05) in baseline characteristics such as gender, age, smoking history, alcohol consumption, tumor location, resection margin status, clinical T stage, or clinical N stage. However, significant differences (p < 0.05) were observed in several pathological outcomes: the NICT group exhibited higher rates of perineural and lymphovascular invasion, more advanced postoperative pathological T and N stages, poorer tumor regression grades (both primary tumor and lymph node), and lower rates of pathological complete response and major pathological response compared to the NCRT group (**Table 2**).

**Table 2:** Comparison of clinical and pathological data between the NCRT and NICT groups.

Variables	Total (n = 346)	NCRT (n=173)	NICT(n=173)	p
Sex, n (%)				1
F	46 (13.3)	23 (13.3)	23 (13.3)	
M	300 (86.7)	150 (86.7)	150 (86.7)	
Age, Mean ± SD	61.1 ± 7.2	61.0 ± 7.0	61.2 ± 7.3	0.852
Smoking, n (%)				0.452
No	84 (24.3)	39 (22.5)	45 (26)	
Yes	262 (75.7)	134 (77.5)	128 (74)	
Drinking, n (%)				0.346
No	102 (29.5)	47 (27.2)	55 (31.8)	
Yes	244 (70.5)	126 (72.8)	118 (68.2)	
Location, n (%)				0.943
Upper	32 (9.2)	16 (9.2)	16 (9.2)	
Middle	129 (37.3)	66 (38.2)	63 (36.4)	
Lower	185 (53.5)	91 (52.6)	94 (54.3)	
Differentiation, n (%)				< 0.001
Well	22 (8.6)	19 (16.7)	3 (2.1)	
Moderately	127 (49.8)	60 (52.6)	67 (47.5)	
Poorly	106 (41.6)	35 (30.7)	71 (50.4)	
Margin, n (%)				0.723
Negative	338 (97.7)	170 (98.3)	168 (97.1)	
Positive	8 (2.3)	3 (1.7)	5 (2.9)	
Nerve invasion, n (%)				< 0.001
No	275 (79.5)	150 (86.7)	125 (72.3)	
Yes	71 (20.5)	23 (13.3)	48 (27.7)	
Vascular invasion, n (%)				< 0.001
No	286 (82.7)	162 (93.6)	124 (71.7)	
Yes	60 (17.3)	11 (6.4)	49 (28.3)	
cT, n (%)				1
2	8 (2.3)	4 (2.3)	4 (2.3)	
3	323 (93.4)	161 (93.1)	162 (93.6)	
4	15 (4.3)	8 (4.6)	7 (4)	
cN, n (%)				0.981
0	13 (3.8)	6 (3.5)	7 (4)	
1	117 (33.8)	60 (34.7)	57 (32.9)	
2	174 (50.3)	86 (49.7)	88 (50.9)	
3	42 (12.1)	21 (12.1)	21 (12.1)	
ypT, n (%)				< 0.001
0	113 (32.7)	75 (43.4)	38 (22)	
1	49 (14.2)	22 (12.7)	27 (15.6)	
2	73 (21.1)	34 (19.7)	39 (22.5)	
3	111 (32.1)	42 (24.3)	69 (39.9)	
ypN, n (%)				0.017
0	215 (62.1)	117 (67.6)	98 (56.6)	
1	84 (24.3)	42 (24.3)	42 (24.3)	
2	40 (11.6)	13 (7.5)	27 (15.6)	
3	7 (2.0)	1 (0.6)	6 (3.5)	
PT-TRG, n (%)				< 0.001
1	104 (30.1)	75 (43.4)	29 (16.8)	
2	67 (19.4)	43 (24.9)	24 (13.9)	
3	103 (29.8)	44 (25.4)	59 (34.1)	
4	72 (20.8)	11 (6.4)	61 (35.3)	
LN-TRG, n (%)				< 0.001
0	200 (57.8)	100 (57.8)	98 (56.6)	
1	21 (6.1)	17 (9.8)	4 (2.3)	
2	28 (8.1)	10 (5.8)	18 (10.4)	
3	53 (15.3)	14 (8.1)	39 (22.5)	
4	44 (12.7)	32 (18.5)	14 (8.1)	
pCR, n (%)				< 0.001
Non-pCR	255 (73.7)	114 (65.9)	141 (81.5)	
pCR	91 (26.3)	59 (34.1)	32 (18.5)	
MPR, n (%)				< 0.001
MPR	171 (49.4)	118 (68.2)	53 (30.6)	
Non-MPR	175 (50.6)	55 (31.8)	120 (69.4)	
Adjuvant therapy,n (%)				1
No	226 (97.0)	116 (96.7)	110 (97.3)	
Yes	7 (3.0)	4 (3.3)	3 (2.7)	

**Figure 2:**
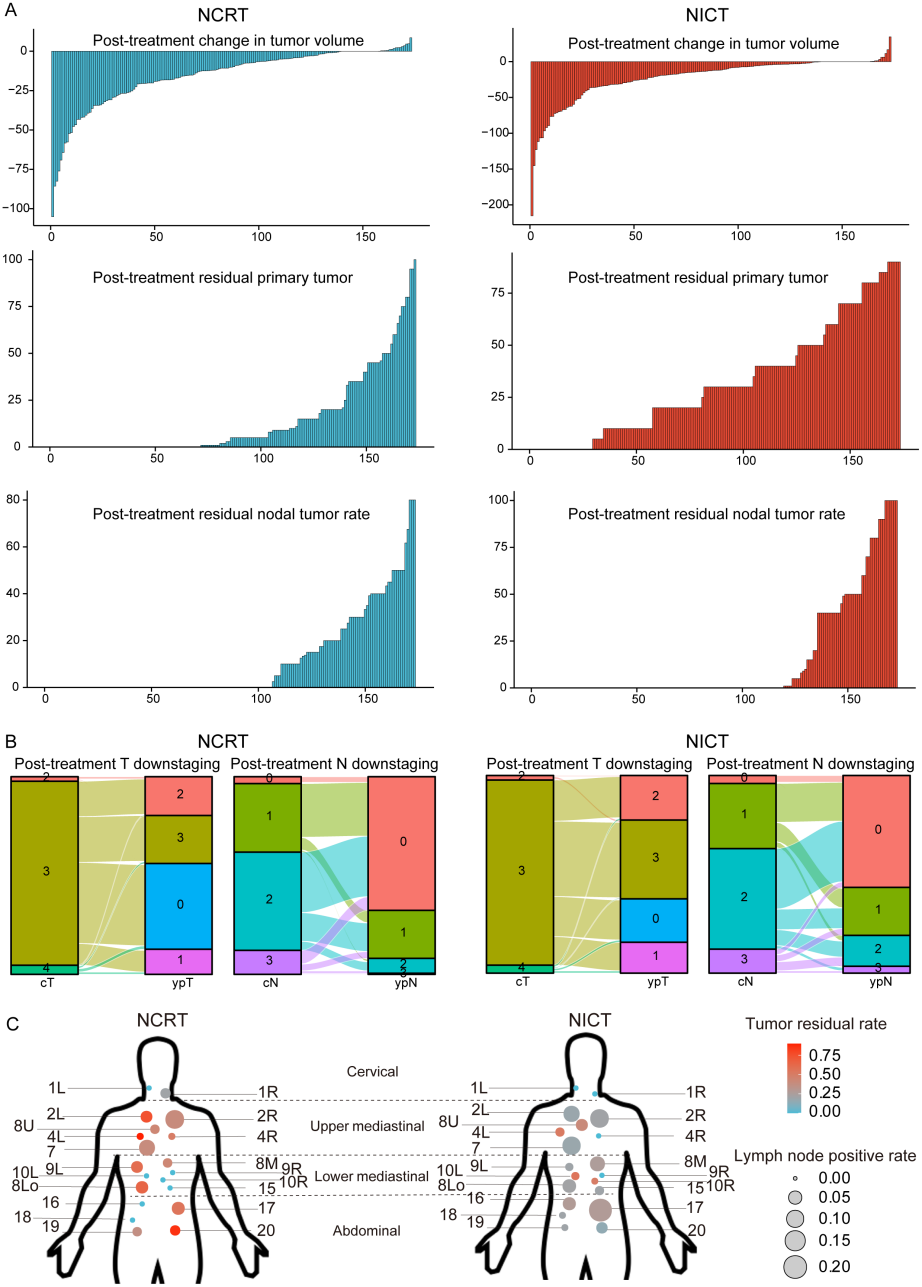
Tumor response following different neoadjuvant therapies (NCRT vs. NICT). **(A)** Changes in tumor volume, residual tumor ratio at the primary site, and lymph node tumor residual ratio after treatment. **(B)** Rates of pathological T category and N category downstaging after treatment. **(C)** Spatial distribution pattern of residual lymph node metastases after treatment, illustrating a “centrifugal” pattern in the NCRT group compared to a more clustered distribution in the NICT group.

A comparative analysis between the NCRT and NICT groups revealed significant differences in treatment response. Tumor volume reduction was greater in the NCRT group (22.09 mm³) compared to the NICT group (15.37 mm³). The residual tumor ratio at the primary site was significantly lower in the NCRT group (14.10%) than in the NICT group (32.23%; p < 0.05). Additionally, the NCRT group demonstrated higher rates of T-stage downstaging (75.7% vs. 54.9%) and Nstage downstaging (83.8% vs. 68.8%; p < 0.05). In contrast, no significant difference was observed in the lymph node tumor residual ratio between the two groups (14.56% in NCRT vs. 11.18% in NICT) (**Figure 2A**). Overall, the NCRT group exhibited greater tumor volume reduction, less primary tumor residue, and higher T/N downstaging rates, although no clear advantage was observed in lymph node tumor clearance (**Figure 2B**). Regarding the distribution of positive lymph nodes, the NCRT group showed a tendency for positive nodes to be located farther from the primary tumor, with fewer positive nodes found in the periesophageal region or within the radiation target volume. In contrast, the NICT group did not exhibit this “centrifugal” distribution pattern (**Figure 2C**).

**Figure 3:**
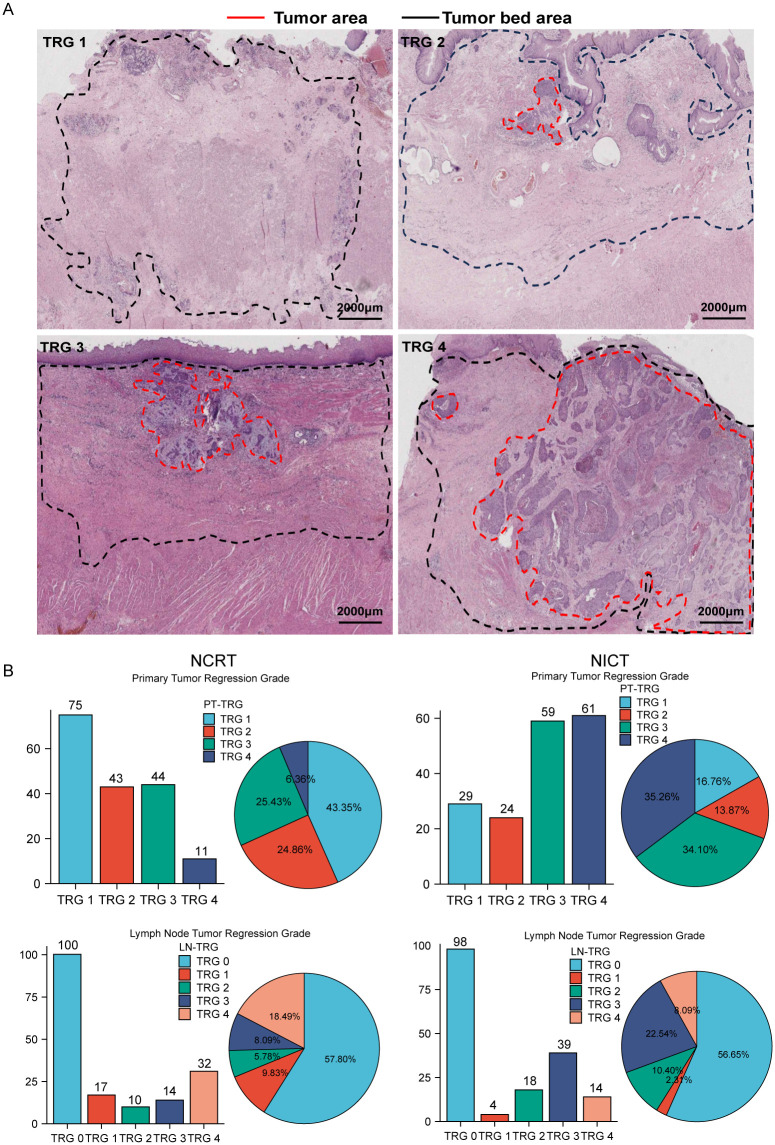
Pathological tumor regression grade (TRG) distribution after neoadjuvant therapy. **(A)** Definition of TRG grades (TRG1–TRG4) for primary tumor and LN-TRG0 for lymph nodes. **(B)** Histograms showing the distribution of primary tumor TRG (upper panel) and lymph node TRG (lower panel) in the NCRT and NICT groups. The NCRT group showed a predominance of low-grade (TRG1–2) primary tumor regression and a bimodal distribution in lymph nodes (peaks at TRG1 and TRG4), while the NICT group exhibited higher-grade primary tumor TRG (TRG3–4) and a centrally clustered lymph node TRG distribution (grades 2–3).

TRG1–4 are defined as follows: TRG1 indicates no residual tumor but presence of a tumor bed; TRG2, <10% residual tumor with marked regression and necrosis; TRG3, 10%–49% residual tumor with partial tumor remaining and focal regressive changes; TRG4, ≥50% residual tumor with extensive residual tumor and minimal regression. LN-TRG0 specifically denotes lymph nodes without tumor and without a tumor bed (**Figure 3A**). In the NCRT group, primary tumor TRG grades were lower, predominantly falling into grades 1–2, whereas the NICT group showed a concentration in grades 3–4. For lymph node TRG, the NCRT group exhibited a bimodal distribution, with most cases clustered at grade 1 or grade 4. In contrast, the NICT group displayed a centrally clustered pattern, with lymph node TRG mainly distributed across grades 2–3 (**Figure 3B**).

**Figure 4:**
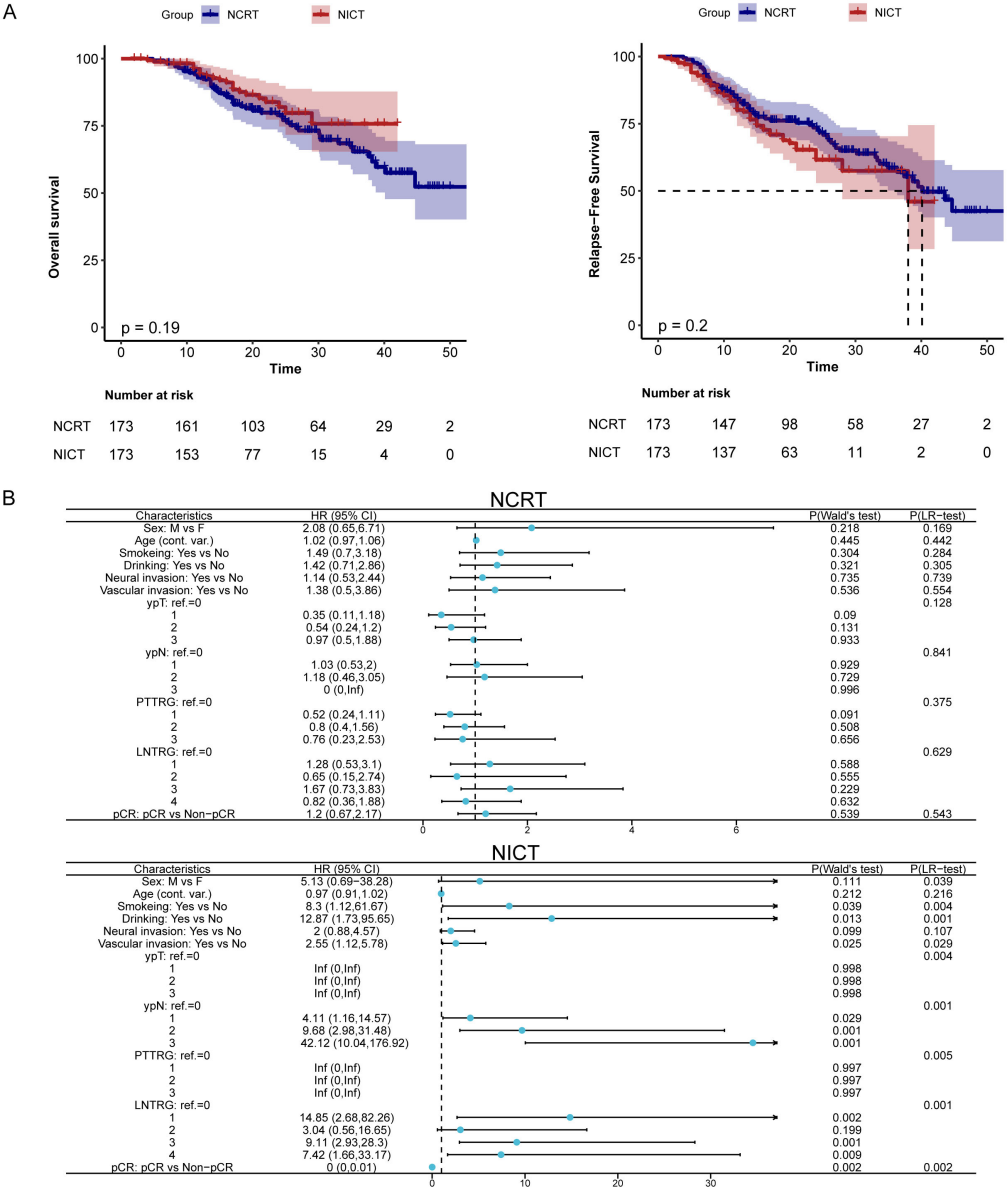
Survival outcomes and prognostic factors. **(A)** Kaplan-Meier curves for overall survival (OS) and recurrence-free survival (RFS) comparing the NCRT and NICT groups (Log-rank p=0.1964). **(B)** Forest plots from multivariable Cox regression analyses showing hazard ratios (HRs) and 95% confidence intervals for clinicopathological factors associated with prognosis in the NCRT and NICT groups separately. In the NICT group, lower LN-TRG and achievement of pCR were significantly associated with improved prognosis (p < 0.05).

The median overall survival (OS) was not reached in either group. The 1-year and 3-year OS rates were 92.9% and 65.4%, respectively, in the NCRT group, compared with 94.1% and 75.8% in the NICT group. Regarding recurrence-free survival (RFS), the median RFS time was 40.1 months (95% CI: 34.9–54.7 months) in the NCRT group and 38.0 months (95% CI: 28.0–42.0 months) in the NICT group. The 1-year and 3-year RFS rates were 82.1% and 48.5% for the NCRT group, versus 76.3% and 41.7% for the NICT group. None of these differences reached statistical significance (Logrank test, p = 0.1964) (**Figure 4A**). Multivariable Cox regression analysis revealed that in the NCRT group, no clinicopathological characteristic—including sex, age, smoking or alcohol history, neural/vascular invasion, ypT stage, ypN stage, PT-TRG, LN-TRG, and pCR status—was significantly associated with prognosis (p ≥ 0.05). In contrast, within the NICT group, a lower LN-TRG and the achievement of pCR were significantly associated with improved prognosis (p < 0.05) (**Figure 4B**).

**Figure 5:**
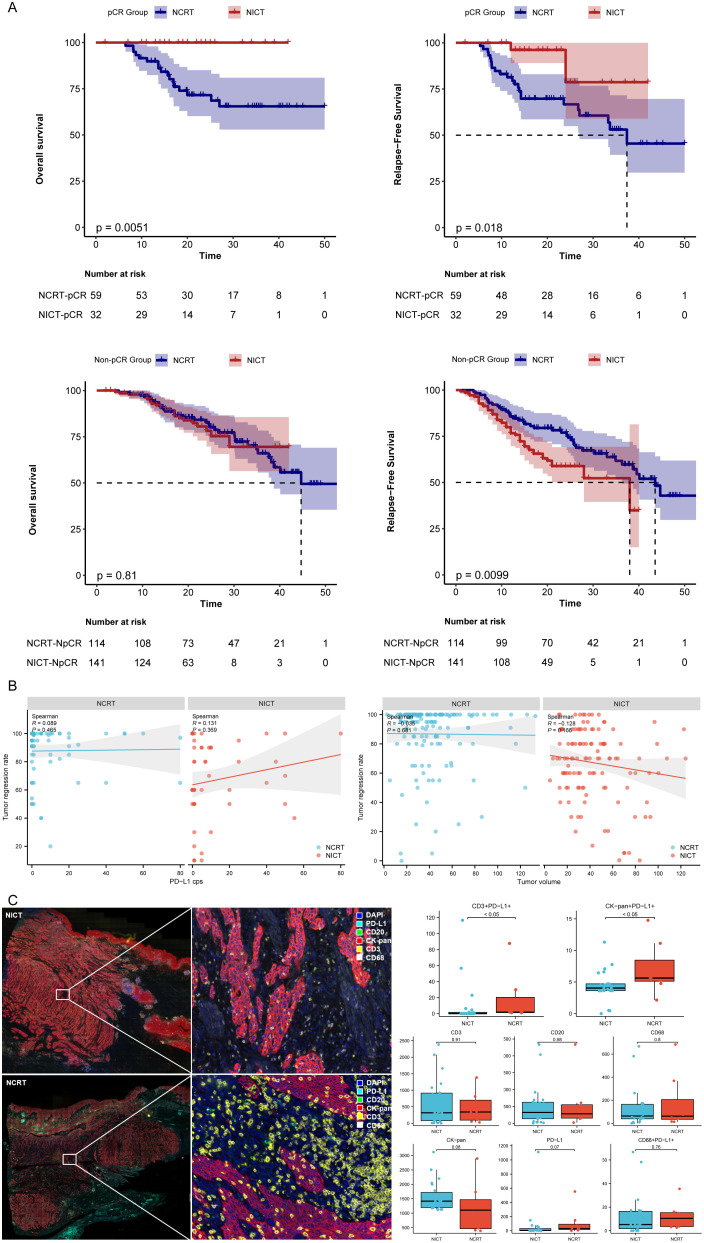
Subgroup survival analysis and tumor microenvironment characterization. **(A)** Kaplan-Meier curves for OS and RFS in the pCR and non-pCR subgroups, stratified by treatment (NCRT vs. NICT). **(B)** Above: Correlation scatter plots showing trends between tumor regression and PD-L1 CPS score or baseline tumor volume in the NICT group. Below: Representative multiplex immunofluorescence images and quantitative analysis comparing post-treatment immune cell densities (CD3^+^, CD20^+^, CD68^+^) and the levels of exhausted T cells (CD3^+^PD-1^+^) and immune-evading tumor cells (CK-Pan^+^PD-L1^+^) between the NCRT and NICT groups.

Subgroup analyses revealed that among patients who achieved a pathological complete response (pCR), those in the NICT-pCR group exhibited significantly superior overall survival (OS, p=0.0051) and recurrence-free survival (RFS, p=0.018) compared to the NCRT-pCR group, indicating that NICT conferred greater long-term survival benefit and lower recurrence risk for pCR patients. In contrast, among patients who did not achieve pCR (non-pCR), there was no significant difference in OS between the two treatment groups (p=0.81). However, the NCRT-non-pCR group demonstrated significantly better RFS than the NICT-non-pCR group (p=0.0099), suggesting that NCRT more effectively reduces the risk of recurrence in this patient population (**Figure 5A**). In the NICT group, tumor regression showed a trend toward positive correlation with PD-L1 CPS score and a negative correlation with baseline tumor volume, suggesting that patients with higher PD-L1 expression and lower tumor burden may achieve more pronounced tumor regression after NICT. This trend was not observed in the NCRT group. Multiplex immunofluorescence analysis revealed no significant differences in the densities of T cells (CD3^+^), B cells (CD20^+^), or macrophages (CD68^+^) within the post-treatment tumor microenvironment between the two groups (p > 0.05). However, the NICT group exhibited significantly lower levels of exhausted T cells (CD3^+^PD-1^+^) and immune-evading tumor cells (CK-Pan^+^PD-L1^+^) compared to the NCRT group after treatment (**Figure 5B**).

## Discussion

In the present study, we conducted a head-to-head comparison of neoadjuvant chemoradiotherapy (NCRT) and neoadjuvant immunochemotherapy (NICT) in a well-balanced cohort of patients with locally advanced esophageal squamous cell carcinoma (ESCC) following 1:1 propensity score matching (PSM). Our findings confirmed that NCRT conferred significantly superior short-term pathological and radiological tumor regression efficacy, whereas no statistically significant differences were observed in long-term overall survival (OS) and recurrence-free survival (RFS) between the two regimens. A striking observation from subgroup analyses was that NICT was associated with markedly improved long-term prognostic outcomes in patients who achieved pathological complete response (pCR), a benefit we postulate is mediated by the remodeling of the tumor immune microenvironment (TIME). In contrast, NCRT provided a distinct advantage in RFS for non-pCR patients. Collectively, these results support a personalized neoadjuvant treatment algorithm for ESCC: NICT should be the preferred strategy for patients with a high predicted likelihood of pCR, while NCRT remains the more favorable option for those in whom pCR is not anticipated.

Neoadjuvant therapy has been firmly established as the standard of care for locally advanced ESCC, with robust evidence from landmark randomized controlled trials such as CROSS and NEOCRTEC5010 demonstrating its ability to induce significant tumor downstaging, improve radical resection rates, and extend patient survival (1, 2). Despite this consensus, the optimal neoadjuvant regimen—NCRT, the long-standing conventional standard, or NICT, the emerging immunotherapy-based alternative—remains a highly contentious clinical issue (12). NCRT is recognized for its potent local tumor ablative effects, while NICT offers well-documented advantages in safety profiles, including fewer treatment-related adverse events, absent radiation-induced tissue adhesion and fibrosis, and thus improved treatment compliance among patients (8, 13). To date, however, few studies have systematically compared the long-term prognostic impacts of these two regimens in a rigorously matched cohort, leaving a critical gap in clinical evidence for treatment selection.

Consistent with the findings of previous comparative studies (14), our results verified that NCRT achieved more pronounced local tumor regression, a lower residual primary tumor ratio, higher T/N downstaging rates, reduced incidences of perineural and lymphovascular invasion, and a higher pCR rate relative to NICT. Notably, this significant short-term advantage in primary tumor response failed to translate into improved long-term survival outcomes. We attribute this phenomenon primarily to the homogenizing effect of R0 radical esophagectomy: the thorough surgical resection of residual tumor tissue and regional lymph nodes may have offset the short-term pathological differences between the two regimens, eliminating the potential survival benefit of superior local tumor regression with NCRT in the curative-intent setting.

With respect to lymph node response, the therapeutic advantage of NCRT was far less pronounced, with no significant difference in the overall residual lymph node tumor rate observed between the NCRT and NICT groups. A key mechanistic insight from our spatial analysis of lymph node involvement was that NCRT induced effective regression of periesophageal lymph nodes adjacent to the primary tumor, a result likely attributable to the precise coverage of these nodes within the radiotherapy target volume (15). Conversely, NCRT exhibited limited efficacy in controlling distant lymph node metastases outside the radiation field. In sharp contrast, NICT mediated relatively uniform and systemic control of lymph node metastases across anatomical sites, with more favorable suppression of distant nodal disease. Given that distant lymph node metastasis is a well-recognized strong adverse prognostic factor in ESCC, the systemic immune-mediated antitumor effect of NICT likely compensates for the superior local ablative effect of NCRT. This complementary therapeutic profile provides a plausible mechanistic explanation for the comparable OS and RFS observed between the two groups, despite their divergent patterns of tumor and lymph node regression.

Multivariate Cox regression analysis further uncovered a divergent prognostic value of treatment response between the two regimens: pCR and a lower lymph node tumor regression grade (LN-TRG) were identified as independent favorable prognostic factors exclusively in the NICT group, with no such associations observed in the NCRT group. Subgroup analyses stratified by pCR status validated this finding: pCR patients treated with NICT had significantly better OS and RFS than those treated with NCRT, while non-pCR patients derived a clear RFS benefit from NCRT. These results highlight a critical distinction in the surrogate value of pCR for long-term survival: pCR serves as a robust and reliable surrogate endpoint for NICT efficacy, whereas it lacks predictive value for prognosis in NCRT-treated patients. This finding has important clinical implications, as it suggests that pCR can be used to guide treatment decision-making and prognostic stratification specifically for patients receiving NICT, but not for those undergoing NCRT.

To further refine personalized treatment selection for NICT, we explored clinical and biological predictors of treatment response in our cohort. Our results revealed that lower baseline tumor burden and higher PD-L1 expression were associated with more significant tumor regression and a higher likelihood of achieving pCR in NICT-treated patients, identifying these as potential predictive biomarkers for NICT efficacy in ESCC. To elucidate the immunological mechanisms underlying this response, we performed multiplex immunofluorescence (mIF) analysis of the TIME, which revealed no significant differences in the densities of core immune cell subsets (CD3^+^ T cells, CD20^+^ B cells, and CD68^+^ macrophages) between the two groups post-treatment. However, the NICT group exhibited significantly reduced proportions of exhausted T cells (CD3^+^PD-1^+^) and immune-evasive tumor cells (CK-Pan^+^PD-L1^+^) compared with the NCRT group. These findings confirm that the therapeutic efficacy of NICT is not mediated by altering the overall density of immune cell infiltration, but rather by reversing T cell exhaustion and abrogating tumor cell immune evasion—key mechanisms that reinvigorate systemic antitumor immunity and enhance long-term anti-tumor surveillance (16). Recent biomarker analyses have likewise linked pretreatment tumor-microenvironment features with pathological response to neoadjuvant chemoimmunotherapy (17). The clinical relevance of sustained immune checkpoint inhibition in resected esophageal cancer is further supported by the disease-free survival benefit observed with adjuvant nivolumab in CheckMate 577 (18).But lack of pre-treatment tissue prevents distinguishing treatment-induced from baseline immune differences—critical for therapy selection. Though PSM balanced clinical factors, baseline immunity was unmeasured, warranting prospective paired-biopsy studies to clarify this and identify biomarkers.

This study has several limitations that warrant acknowledgment and consideration when interpreting our findings. First, despite the use of PSM to minimize selection bias and a relatively large sample size of 346 patients—one of the largest for comparative neoadjuvant ESCC research—the single-center, retrospective study design inherently limits the generalizability of our conclusions to broader patient populations and clinical practice settings. Second, although this study is among the few to report long-term outcomes of NICT in locally advanced ESCC, the median follow-up duration of 24.23 months remains relatively short; longer follow-up is needed to assess late recurrence patterns and confirm the durability of the survival benefit observed in NICT-treated pCR patients. Third, while we characterized key phenotypic features of the TIME using mIF, the underlying molecular mechanisms governing the differential remodeling of the TIME by NCRT and NICT—including changes in immune checkpoint signaling, cytokine profiles, and tumor-stroma crosstalk—were not fully elucidated and merit further investigation. Finally, the present study focused on efficacy and survival outcomes, and did not include a detailed analysis of treatment-related adverse events and postoperative complications; future studies should incorporate these endpoints to provide a more comprehensive risk-benefit assessment of the two regimens. Another limitation is the imbalanced mIF cohort (NCRT: 8 vs. NICT: 30), due to selection bias and financial restrictions. This limits statistical power and generalizability. The observed differences are hypothesis-generating only and require validation in larger, balanced prospective cohorts with paired samples.

In conclusion, the present study provides compelling evidence for divergent therapeutic advantages of NCRT and NICT in locally advanced ESCC: NCRT demonstrates superior local antitumor efficacy, inducing more significant short-term tumor regression and providing a clear RFS benefit for non-pCR patients, while NICT mediates homogeneous systemic control of lymph node metastases, remodels the TIME to reverse immune exhaustion and immune evasion, and confers superior long-term survival outcomes for pCR patients. Our findings further identify high PD-L1 expression and low baseline tumor burden as key factors associated with favorable NICT response, supporting their use to stratify patients for personalized neoadjuvant treatment. For clinical practice, these results advocate for a precision medicine approach: NICT is the preferred neoadjuvant strategy for patients with high PD-L1 expression and low tumor burden (high pCR potential), while NCRT remains a reliable and effective option for all other patients. Future multicenter, prospective randomized controlled trials with longer follow-up are warranted to validate these findings, further elucidate the molecular mechanisms of TIME remodeling, and refine personalized neoadjuvant treatment strategies for locally advanced ESCC.

## Data Availability

The raw data supporting the conclusions of this article will be made available by the authors, without undue reservation, subject to compliance with applicable local laws, regulations, and institutional policies governing data sharing in the authors’ jurisdictions.

## Supplementary material

Supplementary Figure 1 **(A)** An AUC of 0.650 from the ROC curve validates the reliability of the propensity score model. **(B)** Propensity score distribution before and after 1:1 propensity score matching (PSM) and weighting, showing effective balancing of baseline characteristics. **(C)** Standardized mean differences (SMD) of baseline covariates before and after PSM and weighting methods, confirming balanced covariates. **(D)** Schematic illustration of primary tumor regression grade (PT-TRG) and lymph node tumor regression grade (LN-TRG) assessment criteria, defined as the percentage of viable tumor area relative to the total tumor bed area. **(E)** Representative histopathological images of LN-TRG 1–4, showing the extent of residual viable tumor (red dots) within the tumor bed (black dashed outline).
